# Study on Preparation of Ultra-High-Molecular-Weight Polyethylene Pipe of Good Thermal-Mechanical Properties Modified with Organo-Montmorillonite by Screw Extrusion

**DOI:** 10.3390/ma13153342

**Published:** 2020-07-27

**Authors:** Zhouchao Guo, Rui Xu, Ping Xue

**Affiliations:** School of Mechanical and Electrical Engineering, Beijing University of Chemical Technology, Beijing 100029, China; gzctaopu@yeah.net (Z.G.); xuruibuct@163.com (R.X.)

**Keywords:** ultra-high-molecular-weight polyethylene, organo-montmorillonite, modified pipe, screw extrusion

## Abstract

The study of processing characteristic and property optimization of ultra-high-molecular-weight polyethylene (UHMWPE) pipe is increasingly performed, mainly focusing on difficulties in the melting process and poor thermal-mechanical properties after forming, which have limited the wider engineering application of UHMWPE pipe. In this study, organo-montmorillonite (OMMT)-modified UHMWPE pipe with good thermal-mechanical properties was prepared by screw extrusion molding. First, high-density polyethylene was subjected to fluidity modification so that the screw extrusion molding of UHMWPE pipe was feasible. Then, OMMT-modified UHMWPE pipes under different addition amounts of OMMT were innovatively prepared by extrusion. Furthermore, the effects of the addition amounts of the compatibilizer HDPE-g-MAH and the silane coupling agent γ-(2,3-epoxy propoxy) propyl trimethoxy silane (KH560) on the thermal properties of OMMT-modified UHMWPE pipe were investigated for the first time. Compared with those of pure UHMWPE pipe, the Vicat softening temperature (from 128 to 135.2 °C), thermal deformation temperature (from 84.4 to 133.1 °C), bending strength (from 27.3 to 39.8 MPa), and tensile strength (from 20.8 to 25.1 MPa) of OMMT-modified UHMWPE pipe were greatly increased. OMMT-modified UHMWPE pipe with good thermal-mechanical properties was able to be prepared by extrusion for the first time. The compatibilizer method of HDPE-g-MAH was slightly more effective than the coupling agent method of KH560.

## 1. Introduction 

Ultra-high-molecular-weight polyethylene (UHMWPE) is a polymer that has been used in pipelines because of its good physicochemical and tribological properties [1,2]. Nowadays, UHMWPE is mainly prepared from synthesis by the Ziegler catalyst method. According to existing literature, UHMWPE prepared by the Ziegler catalyst method is known for three characteristics: high viscosity, low coefficient of friction, and low critical shear rate [3,4]. The study of processing characteristic and property optimization of UHMWPE pipe is increasingly performed, mainly focusing on difficulties in the melting process and poor thermal-mechanical properties after forming, which have limited the wider engineering application of UHMWPE pipe.

Compression molding without flow, which features simple operation and low cost, can overcome the disadvantage of the high viscosity of UHMWPE [5,6,7,8,9]. However, low production efficiency and low-quality consistency limit the practical application of compression molding. Smith [10] has proposed a new method to improve the production efficiency of compression molding. Expander is added for the pre-disentanglement of molecular chains of UHMWPE; however, the production efficiency of compression molding is still not high enough.

Screw extrusion molding, which features continuous production, can overcome the disadvantage of the low production efficiency of compression molding [11]. By the fluidity modification of UHMWPE, screw extrusion molding of UHMWPE can be feasible. Fluidity modification methods of UHMWPE mainly include blending and using rheological modifiers. For the blending method, UHMWPE is usually blended with resins that have good fluidity, such as low-density polyethylene [12,13], high-density polyethylene [14,15,16], and polypropylene [12,17]. For the rheological modifier method, the addition of a rheological modifier can decrease the entanglement of molecular chains of UHMWPE and plays the role of lubricant [18,19].

However, the fluidity modification of UHMWPE will inevitably induce a degree of decrease in thermal and mechanical properties. Thus, the strengthening modification of UHMWPE is necessary for good thermal-mechanical properties. Strengthening modification methods of UHMWPE mainly include physical modification [20,21,22], chemical modification [23,24,25], and filled composite modification [26,27]. For the filled composite modification, both physical and chemical methods are used. In addition, a variety of techniques are used to improve the interaction between matrix and filler. However, the screw extrusion molding of OMMT-modified UHMWPE is rarely reported in existing literature, and OMMT-modified UHMWPE pipe with good thermal-mechanical properties is still new.

In this study, the fluidity modification of UHMWPE was performed so that the screw extrusion molding of UHMWPE pipe was feasible. Then, with different addition amounts of organo-montmorillonite (OMMT), OMMT-modified UHMWPE pipes were innovatively prepared in a single screw extruder. In addition, the effects of the addition amounts of HDPE-g-MAH and KH560 on the thermal properties of OMMT-modified UHMWPE pipe were investigated for the first time. With the management of thermal and mechanical properties, the Vicat softening temperature, thermal deformation temperature, bending strength, and tensile strength of OMMT-modified UHMWPE pipe were greatly increased. OMMT-modified UHMWPE pipe with good thermal-mechanical properties could be prepared by extrusion for the first time.

## 2. Preparation of OMMT-Modified UHMWPE Pipe Samples

### 2.1. Materials

UHMWPE (granule/GUR4152) was supplied by Ticona Co., Ltd., Frankfurt, Germany. OMMT (powder/I.44P), used as filler, was provided by Nanocor Co., Ltd., Arlington Heights, IL, USA. HDPE (powder/5070), used for the fluidity modification of UHMWPE, was provided by Beijing Yanshan Petrochemical High-Tech Co., Ltd., Beijing, China. HDPE 5070 was chosen because the melt flow index (MFI) of this HDPE is high (6.1~8.0). HDPE-g-MAH (powder/ME5506), used as a compatibilizer, was obtained from Nantong Rizhisheng New Polymer Material Technology Co., Ltd., Nantong, China. A silane coupling agent (liquid/KH560), used to improve the interaction between UHMWPE/HDPE and OMMT, was supplied by Hangzhou Jiexika Chemical Co., Ltd., Hangzhou, China. The chosen silane should have both an inorganic reactive group and an organic reactive group so that it can enhance the interaction between the filler OMMT (inorganic phase) and the matrix UHMWPE/HDPE (organic phase). Ethanol (liquid) was supplied by Dongguan Jintai Chemical Technology Co., Ltd., Dongguan, China.

### 2.2. Experimental Equipment

A single screw extruder (with a screw diameter of 45 mm), used for ultra-high-molecular-weight material, was developed by Beijing University of Chemical Technology, China. A high-speed mixer (SHR-25A) was provided by Zhangjiagang Yongli Mechanical Co., Ltd., Zhangjiagang, China. An electric constant temperature drying oven (SFG-02.400) was provided by Hengfeng Medical Device Co., Ltd., Huangshi, China.

### 2.3. Preparation of OMMT-Modified UHMWPE Pipe Samples

The preparation of OMMT-modified UHMWPE pipe samples is summarized as follows:

Step 1. The mixtures of materials were prepared with a compatibilizer method and a coupling agent method.

(a) For the compatibilizer method, the OMMT was kept in a drying oven at a constant temperature of 110 °C for four hours. Then, UHMWPE, HDPE, OMMT, HDPE-g-MAH, and other additives were added into a mixer for homogeneous mixing.
(b) For the coupling agent method, a solution of KH560 and ethyl alcohol was prepared with a volume ratio of 1:10. Then, the solution was premixed uniformly with OMMT, and the mixture was kept in a drying oven at a constant temperature of 110 °C for four hours. Lastly, UHMWPE, HDPE, OMMT, KH560, and other additives were added into a mixer for homogeneous mixing.

The mixer has two levels of revolving speed: 725 and 1440 rpm. At room temperature, the mixer works at 725 rpm for five minutes and then at 1440 rpm for another five minutes.

**Figure 1:**
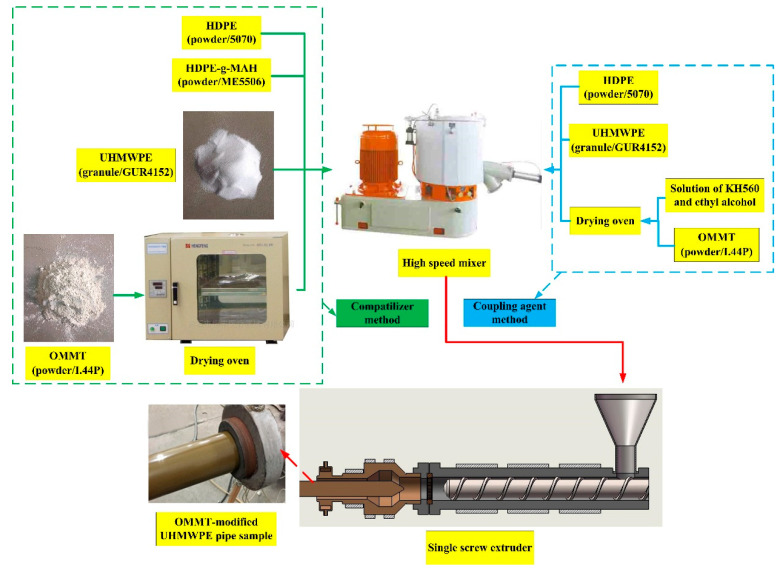
Flowchart of preparation of organo-montmorillonite (OMMT)-modified ultra-high-molecular-weight polyethylene (UHMWPE) pipe samples.

Step 2. The mixtures were processed into pipe samples (see Figure 1) in a single screw extruder. The process parameters of screw extrusion molding are shown in Table 1.

### 2.4. Characterization of OMMT-Modified UHMWPE Pipe

#### 2.4.1. Vicat Softening Temperature and Thermal Deformation Temperature

A tester of Vicat softening temperature and thermal deformation temperature (KXRW-300CL-3, Chengde Taiding Tester Manufacturing Co., Ltd., Chengde, China) was used to test the Vicat softening temperature and thermal deformation temperature of OMMT-modified UHMWPE pipe according to China national standard GB/T1633-2000. The lengths, widths, and thicknesses of the sample bars were 10 × 10 × 4 mm^3^ for the Vicat softening temperature and 80 × 10 × 4 mm^3^ for the thermal deformation temperature. The pressure 0.45 MPa was used to test the thermal deformation temperature. The sample bars were obtained from pipe samples by cutting and flattening according to China national standards (the same below).

#### 2.4.2. Shore Hardness

A Shore D durometer (BS61, ZWICK Co., Ltd., Ulm, Germany) was used to test the Shore hardness of OMMT-modified UHMWPE pipe according to China national standard GB/T2411-2008. The length, width, and thickness of the sample bar were 50 × 50 × 4 mm^3^.

#### 2.4.3. SEM

SEM (Hitachi S-4700, Hitachi Co., Ltd., Tokyo, Japan) was used to analyze the microstructures of OMMT-modified UHMWPE pipe. Cooled with liquid nitrogen, the sample was sprayed with gold after a brittle fracture.

#### 2.4.4. DSC

DSC (TA-Q100, TA Co., Ltd., Pennsylvania, PA, USA) was used to measure the melting enthalpy, melting point, and crystallinity of OMMT-modified UHMWPE pipe. Under the protection of nitrogen gas, each sample of 5 milligrams was heated from room temperature to 200 °C at a rate of 10 °C/min and then cooled to room temperature.

#### 2.4.5. Bending Strength and Tensile Strength

A universal material testing machine (KXWW, Chengde Taiding Tester Manufacturing Co., Ltd., Chengde, China) was used to test the bending strength and tensile strength of OMMT-modified UHMWPE pipe according to China national standards GB/T9341-2000 and GB/T1040-2006. The lengths, widths, and thicknesses of the sample bars were 80 × 10 × 4 mm^3^ for the bending strength and 120 × 10 × 4 mm^3^ for the tensile strength.

#### 2.4.6. Small-Angle X-ray Diffraction

An X-ray diffractometer (2500VB2+PC, RIGAKU, Tokyo, Japan) was used to measure the diffraction angle at the diffraction peak of OMMT-modified UHMWPE pipe. The diffraction test was performed under CuKα-ray radiation with a tube voltage of 40 kV, a tube current of 50 mA, and a scanning speed of 5°/min.

## 3. Thermal-Mechanical Property Management

Firstly, the thermal and mechanical properties of pure UHMWPE pipe were tested and are shown in Table 2. The equipment and methods used to determine the properties in Table 2 were the same as those used to characterize OMMT-modified UHMWPE pipe in Section 2.4. Then, during our experiments and tests, the effects of the addition amounts of filler, compatibilizer, and coupling agent on the thermal and mechanical properties of OMMT-modified UHMWPE pipe were studied.

### 3.1. Addition Amount of OMMT

In order to study the effect of the addition amounts of the filler OMMT on the thermal and mechanical properties of UHMWPE pipe, OMMT-modified UHMWPE pipes were prepared using different addition amounts of OMMT. The other additives in Table 3 are the processing aids calcium stearate and zinc stearate.

**Figure 2:**
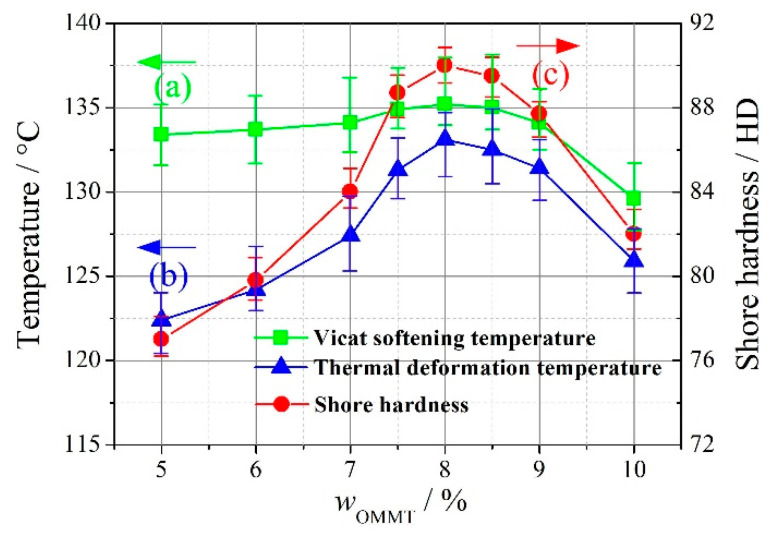
The heat resistance and hardness changes of OMMT-modified UHMWPE with increasing amounts of OMMT. (**a**) Vicat softening temperature, (**b**) thermal deformation temperature, (**c**) Shore hardness (repeated three times).

As shown in Figure 2, the Vicat softening temperature, thermal deformation temperature, and Shore hardness show the same trend and increase with an increasing amount of OMMT for *w*_OMMT_ ≤ 8%. When the nano-filler OMMT is introduced, it disperses in the matrix of UHMWPE/HDPE. With an increasing amount of OMMT, more polymer macromolecules should be adsorbed on the surface of the nano-filler OMMT. As a result, UHMWPE/HDPE entanglements will be strengthened. The strengthened entanglements will enhance the intermolecular forces and the rigidity of the OMMT-modified UHMWPE. According to its testing principle, the Vicat softening temperature can be regarded as the hardness of materials under heating conditions, i.e., the rigidity of materials. Therefore, the Vicat softening temperature increases with an increasing amount of OMMT for *w*_OMMT_ ≤ 8%. When the OMMT-modified UHMWPE is heated, OMMT around the molecular chains of UHMWPE/HDPE plays the role of a physical cross-link, which limits the thermal motion of UHMWPE/HDPE molecular chains. Therefore, the thermal deformation temperature increases with increasing amounts of OMMT for *w*_OMMT_ ≤ 8%. Moreover, with the filling of OMMT, the OMMT-modified UHMWPE is denser in molecular chain distribution, more complex in space structure, and more difficult to invade than the matrix UHMWPE/HDPE. Therefore, the Shore hardness increases with increasing amounts of OMMT for *w*_OMMT_ ≤ 8%.

In Figure 2, it can also be seen that the Vicat softening temperature, thermal deformation temperature, and Shore hardness first increase, reach their maximums at *w*_OMMT_ = 8%, and then decrease. Therefore, a reasonable addition amount of OMMT is 8%. At *w*_OMMT_ = 8%, the Vicat softening temperature, thermal deformation temperature, and Shore hardness are 135.2 °C, 133.1 °C, and 90 HD, respectively.

**Figure 3:**
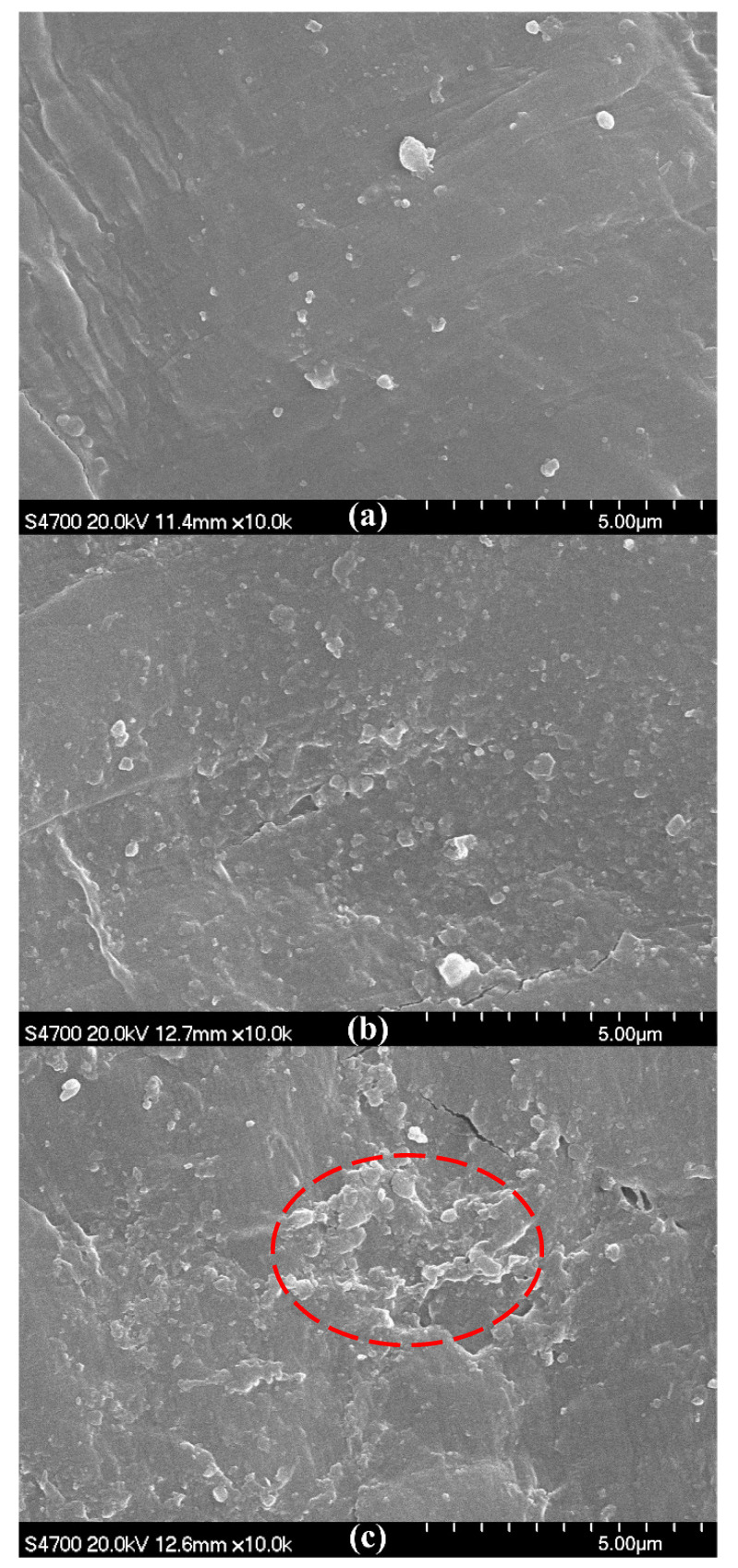
SEM micrographs of fracture surfaces of OMMT-modified UHMWPE pipes. (**a**) *w*_OMMT_ = 5%, (**b**) *w*_OMMT_ = 8%, (**c**) *w*_OMMT_ = 10%.

To study the deterioration of the Vicat softening temperature, thermal deformation temperature, and Shore hardness, SEM micrographs of the fracture surfaces at *w*_OMMT_ = 5%, 8%, and 10% were compared, as shown in Figure 3. The SEM micrographs show that no or slight aggregation of OMMT is found at *w*_OMMT_ = 5% and 8%, while aggregation of OMMT is found at *w*_OMMT_ = 10%. At *w*_OMMT_ = 8%, OMMT disperses uniformly in the matrix UHMWPE/HDPE without aggregation; however, excessive amounts of OMMT give rise to aggregation, which can be a material defect and weaken the thermal and mechanical properties of OMMT-modified UHMWPE. In Figure 2, the Vicat softening temperature, thermal deformation temperature, and Shore hardness decrease with increasing amounts of OMMT for *w*_OMMT_ > 8%.

**Figure 4:**
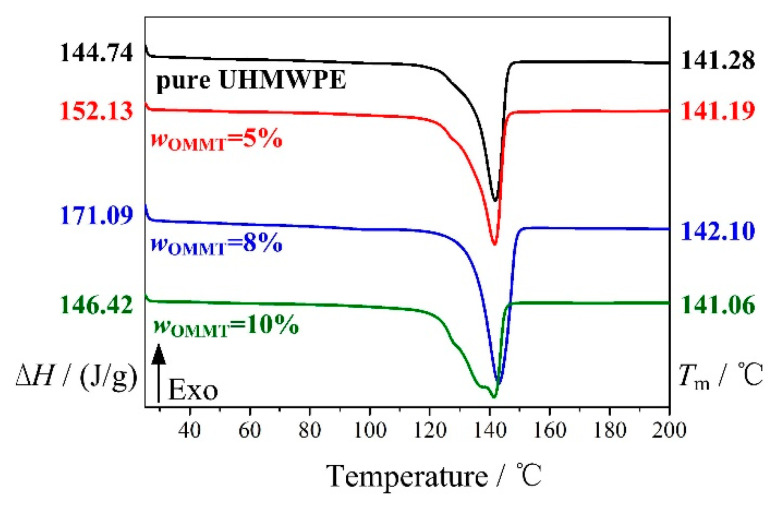
DSC curves of OMMT-modified UHMWPE under different addition amounts of OMMT.

**Figure 5:**
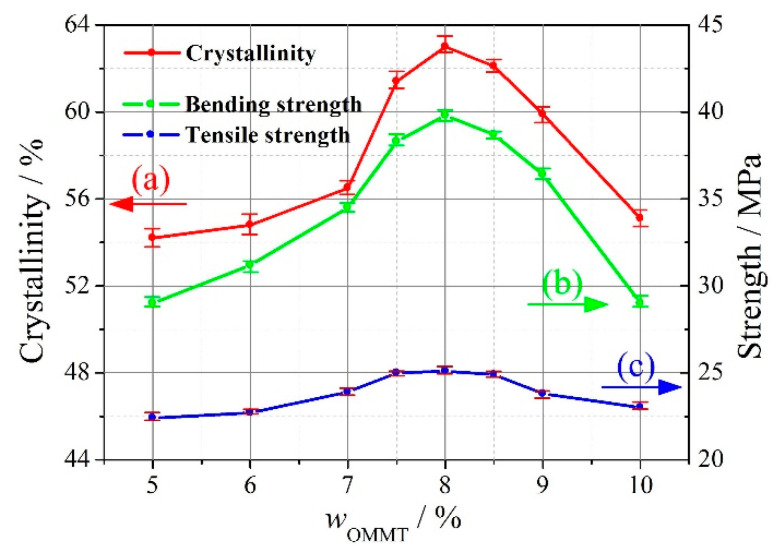
The crystallinity, bending strength, and tensile strength of the OMMT-modified UHMWPE with increasing amounts of OMMT. (**a**) Crystallinity, (**b**) bending strength, (**c**) tensile strength (repeated three times).

In addition, Figure 4 shows the DSC curves of the OMMT-modified UHMWPE at *w*_OMMT_ = 5%, 8%, and 10%. The melting enthalpy (Δ*H*) and melting point (*T*_m_) of the OMMT-modified UHMWPE, which can be obtained from the DSC curves, are also shown on the left and right sides in Figure 4, respectively. With Δ*H* and the melting enthalpy of fully crystallized UHMWPE (Δ*H*_0_) known, the crystallinity (*X*_c_) of the OMMT-modified UHMWPE can be obtained by the following equation and as shown in Figure 5a:

(1) $$X_{c}=\Delta H/(\Delta H_{0}\times w_{\mathrm{UHMWPE}})\times 100\%$$

It is found that the crystallinity of the OMMT-modified UHMWPE first increases, reaches a maximum at *w*_OMMT_ = 8%, and then decreases with increasing amounts of OMMT. For *w*_OMMT_ ≤ 8%, the addition of OMMT gives rise to the heterogeneous nucleation crystallization of UHMWPE/HDPE, which increases the crystallinity and the melting point of OMMT-modified UHMWPE; however, for *w*_OMMT_ > 8%, an excessive amount of OMMT results in aggregation, which will weaken the effect of the heterogeneous nucleation crystallization of UHMWPE/HDPE. It is also found that the bending and tensile strengths of the OMMT-modified UHMWPE have the same trend as the crystallinity (refer to Figure 5b,c) with an increasing amount of OMMT. At *w*_OMMT_ = 8%, the crystallinity, bending strength, and tensile strength are 63%, 39.8 MPa, and 25.1 MPa, respectively.

**Figure 6:**
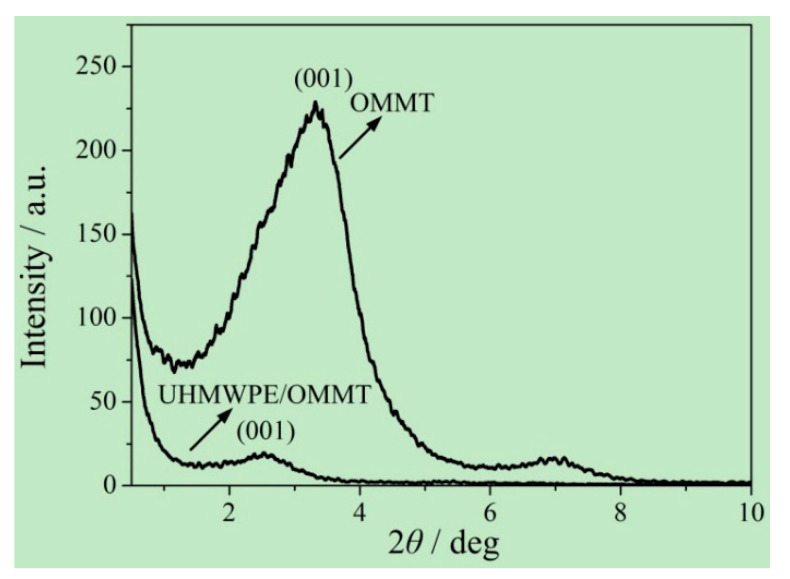
Small-angle XRD curves of OMMT and OMMT-modified UHMWPE.

To better understand the changes in the bending and tensile strengths, small-angle X-ray diffraction (XRD) curves of OMMT and OMMT-modified UHMWPE (*w*_OMMT_ = 8%) were compared, as shown in Figure 6. It can be seen that the diffraction peaks of OMMT at the crystal (001) for OMMT and OMMT-modified UHMWPE (*w*_OMMT_ = 8%) occur at 2*θ* = 3.314 and 2.538 deg, respectively. Then, the layer distances can be obtained by the Bragg equation:

(2) 2dsinθ=nλ

and the calculated layer distances for OMMT and OMMT-modified UHMWPE (*w*_OMMT_ = 8%) are 2.663 and 3.477 nm, respectively.

Compared with that of OMMT, the diffraction peak for the crystal (001) of the OMMT-modified UHMWPE (*w*_OMMT_ = 8%) moves to a smaller angle, which indicates that the molecular chains of UHMWPE/HDPE enter the lamellar structure of OMMT. With more molecular chains of UHMWPE/HDPE entering the lamellar structure of OMMT, the OMMT-lamellar-coated molecule chains of UHMWPE/HDPE are able to sustain larger bending loads. In addition, under tension loads, the molecular chains of UHMWPE/HDPE in the lamellar structure of OMMT extend along the lamellar structure, which gives rise to a uniform distribution of tensile stress. As a result, both the bending and tensile strengths of the OMMT-modified UHMWPE increase with an increasing amount of OMMT for *w*_OMMT_ ≤ 8%. However, for *w*_OMMT_ > 8%, an excessive amount of OMMT induces aggregation, which will be a material defect and weaken the effect of OMMT-lamellar-coated molecular chains.

### 3.2. Addition Amount of HDPE-g-MAH

As shown in Table 4, to study the effect of the addition amount of HDPE-g-MAH on the thermal properties of OMMT-modified UHMWPE pipe, OMMT-modified UHMWPE with different addition amounts of HDPE-g-MAH was prepared. The other additives in Table 4 are the processing aids calcium stearate and zinc stearate.

**Figure 7:**
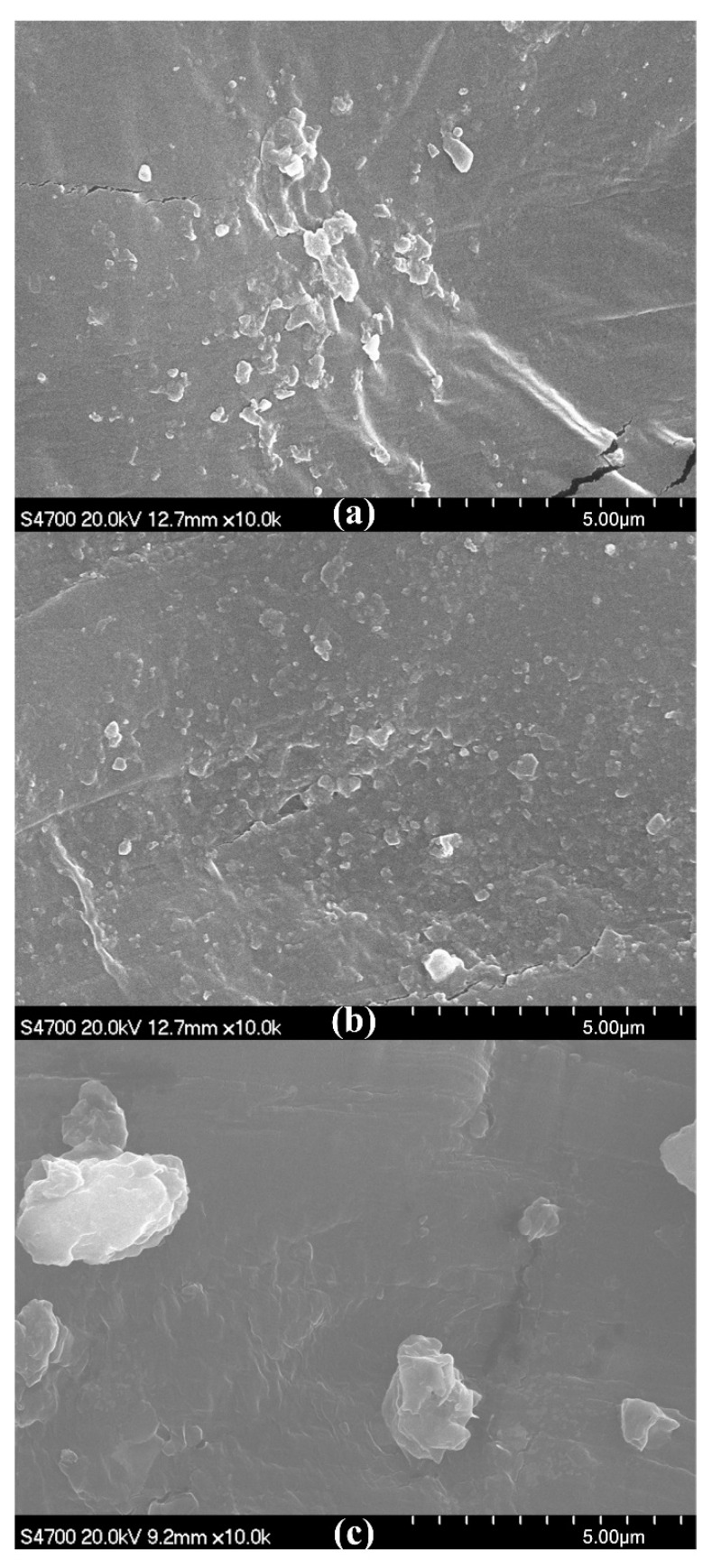
SEM micrographs of fracture surfaces of OMMT-modified UHMWPE pipes. (**a**) *w*_HDPE-g-MAH_ = 1%, (**b**) *w*_HDPE-g-MAH_ = 3%, (**c**) *w*_HDPE-g-MAH_ = 5%.

**Figure 8:**
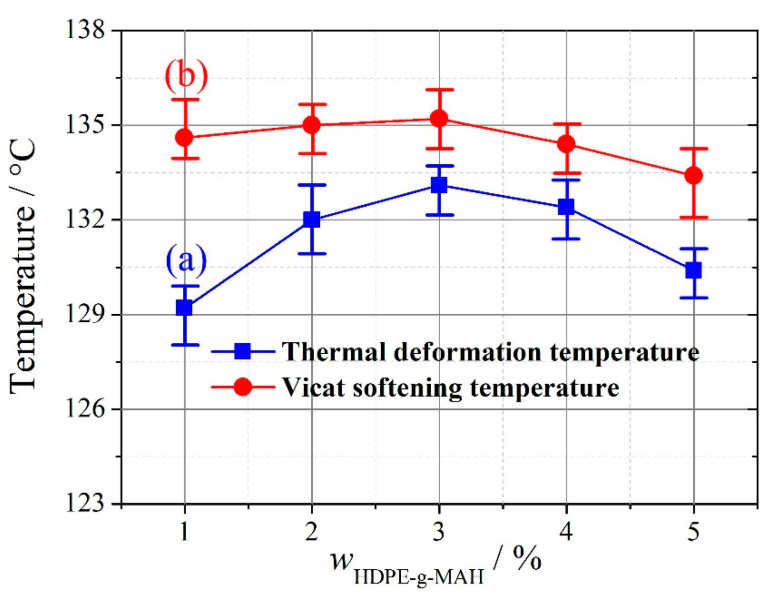
Thermal deformation temperature and Vicat softening temperature of OMMT-modified UHMWPE with increasing amounts of HDPE-g-MAH. (**a**) Thermal deformation temperature, (**b**) Vicat softening temperature (repeated three times).

As shown in Figure 7a, slight aggregation of OMMT occurs at *w*_HDPE-g-MAH_ = 1% because HDPE-g-MAH at the mass fraction 1% is not enough to enable OMMT at the mass fraction 8% to interact fully with the matrix UHMWPE/HDPE. In Figure 7b, no aggregation of OMMT can be seen, and OMMT disperses uniformly; however, with further increases of the addition amount of HDPE-g-MAH, aggregation of OMMT is seen in Figure 7c. When an excessive amount of HDPE-g-MAH is added, HDPE-g-MAH agglomeration can be induced. In this situation, the compatibilizer HDPE-g-MAH not only does not strengthen the interaction between OMMT and UHMWPE/HDPE but can also be a material defect. Therefore, aggregation of OMMT is seen in Figure 7c. For macroscopic thermal parameters, as shown in Figure 8, both thermal deformation temperature and Vicat softening temperature first increase, reach their maximums at *w*_HDPE-g-MAH_ = 3%, and then decrease with increasing addition amounts of HDPE-g-MAH. Thus, at *w*_OMMT_ = 8%, a reasonable addition amount of HDPE-g-MAH is 3%.

### 3.3. Addition Amount of KH560

In addition, KH560 was used as a coupling agent for the pretreatment of OMMT. To study the effect of the addition amount of KH560 on the thermal properties of OMMT-modified UHMWPE, OMMT-modified UHMWPE with different addition amounts of KH560 (as shown in Table 5) was prepared. The other additives in Table 5 are the processing aids calcium stearate and zinc stearate.

**Figure 9:**
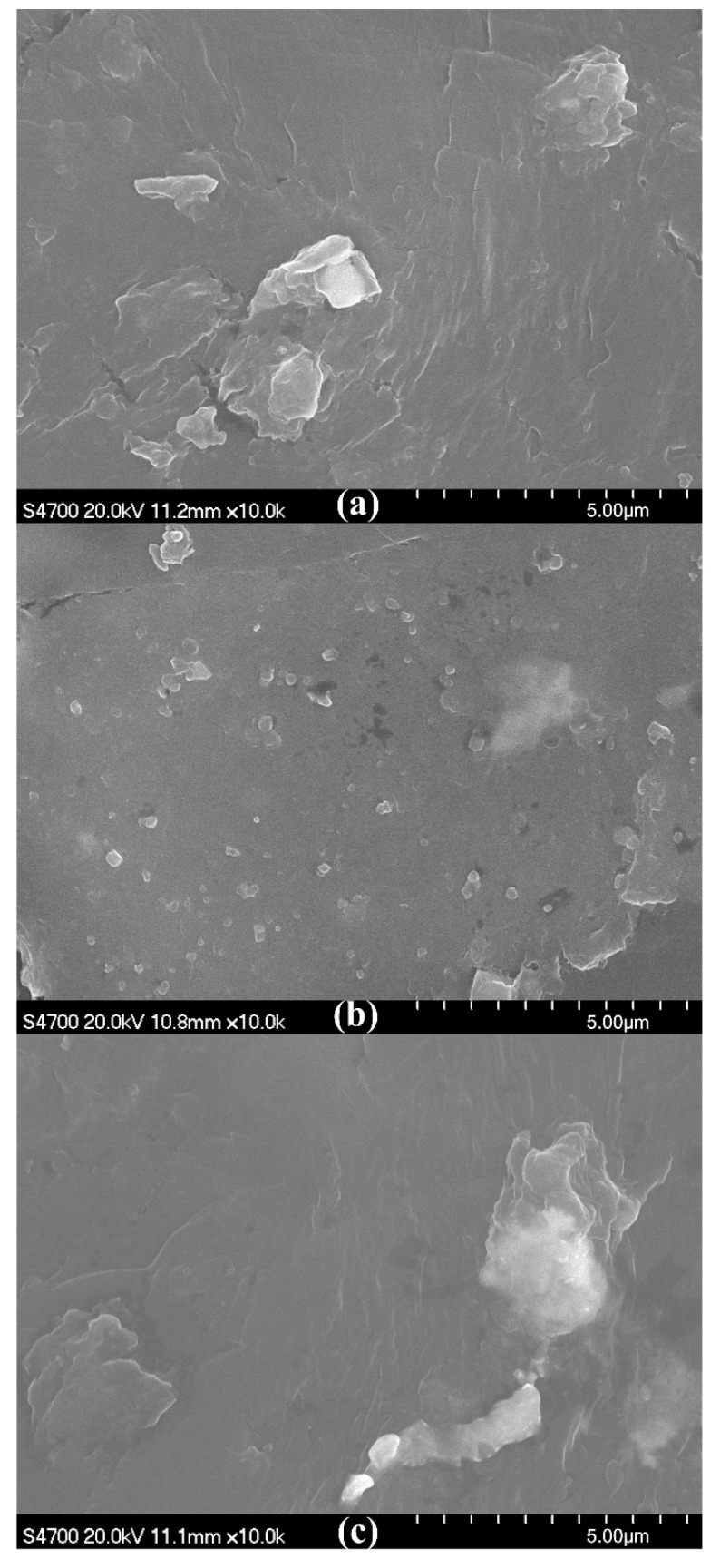
SEM micrographs of fracture surfaces of OMMT-modified UHMWPE. (**a**) *w*_KH560_/*w*_OMMT_ = 0.5%, (**b**) *w*_KH560_/*w*_OMMT_ = 1%, (**c**) *w*_KH560_/*w*_OMMT_ = 1.5%.

**Figure 10:**
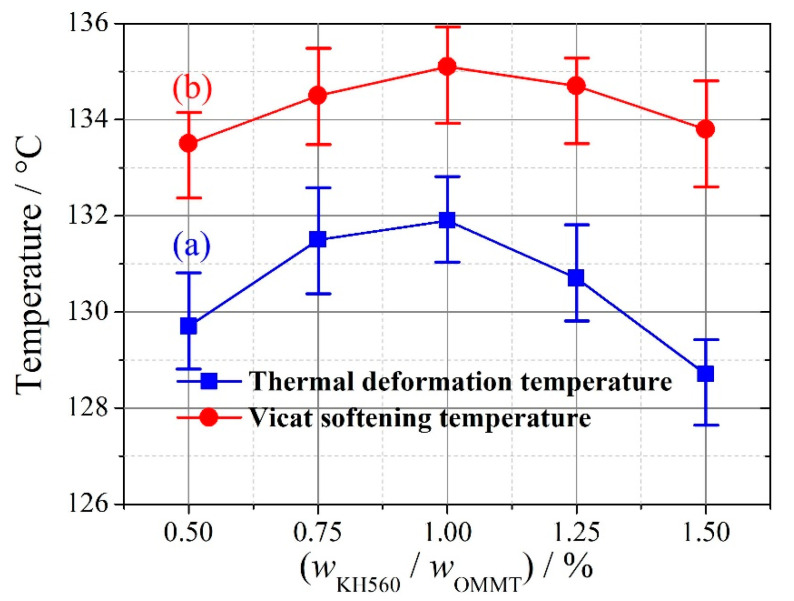
Thermal deformation temperature and Vicat softening temperature of OMMT-modified UHMWPE with an increasing amount of KH560. (**a**) Thermal deformation temperature, (**b**) Vicat softening temperature (repeated three times).

At *w*_KH560_/*w*_OMMT_ = 0.5%, aggregation of OMMT occurs, as shown in Figure 9a, because the amount of KH560 is not high enough to enable OMMT at the mass fraction 8% to interact fully with the matrix UHMWPE/HDPE. In Figure 9b, no aggregation of OMMT can be seen, and OMMT disperses uniformly; however, with further increases of the addition amount of KH560, aggregation of OMMT is seen in Figure 9c because an excessive amount of KH560 will induce a weak interphase film and weaken the interaction between OMMT and UHMWPE/HDPE. For macroscopic thermal parameters, both thermal deformation temperature and Vicat softening temperature first increase, reach their maximums at *w*_KH560_/*w*_OMMT_ = 1%, and then decrease with an increasing addition amount of KH560, as shown in Figure 10. Thus, at *w*_OMMT_ = 8%, a reasonable addition amount of KH560 is *w*_KH560_/*w*_OMMT_ = 1%.

A further comparison between Figure 7b and Figure 9b shows that OMMT disperses more uniformly in Figure 7b than in Figure 9b, an indication that both thermal deformation temperature and Vicat softening temperature are higher by using HDPE-g-MAH than by using KH560. It can be seen from Figure 8 and Figure 10 that the thermal deformation temperature and Vicat softening temperature are 1.2 and 0.1 °C higher by using HDPE-g-MAH (*w*_HDPE-g-MAH_ = 3%) than by using KH560 (*w*_KH560_/*w*_OMMT_ = 1%).

**Figure 11:**
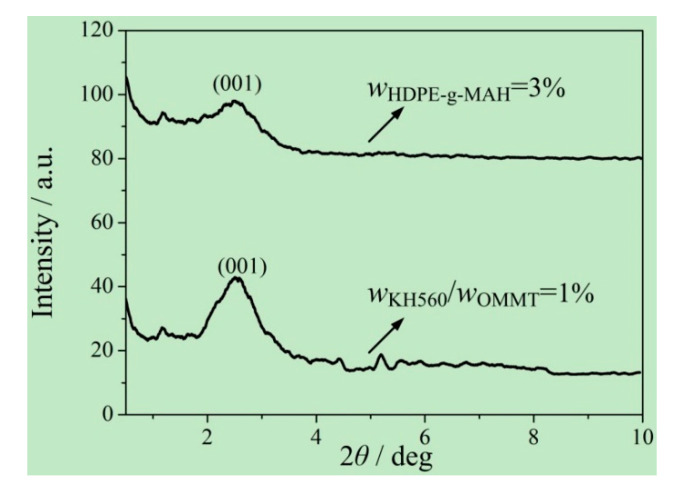
Comparison of small-angle XRD curves of OMMT-modified UHMWPE between using HDPE-g-MAH and using KH560.

To better understand the differences in thermal properties between using HDPE-g-MAH (*w*_HDPE-g-MAH_ = 3%) and using KH560 (*w*_KH560_/*w*_OMMT_ = 1%), small-angle XRD curves of the corresponding OMMT-modified UHMWPE were compared, as shown in Figure 11. It can be seen that the diffraction peaks of OMMT at the crystal face (001) for the corresponding OMMT-modified UHMWPE occur at 2*θ* = 2.538 and 2.584 deg, respectively. The layer distances of OMMT for the corresponding OMMT-modified UHMWPE can be obtained by Equation (2), and the calculated layer distances are 3.477 and 3.415 nm, respectively. A longer layer distance by using HDPE-g-MAH than by using KH560 indicates that more molecular chains of UHMWPE/HDPE enter the lamellar structure of OMMT. The distance between molecular chains is shorter in the lamellar structure than outside the lamellar structure. With more molecular chains in the lamellar structure, the intermolecular force becomes stronger. Thus, the strong intermolecular force limits the thermal motion of molecular chains of UHMWPE/HDPE and increases the thermal deformation temperature and Vicat softening temperature.

## 4. Conclusions

Based on related experiments and tests, OMMT-modified UHMWPE pipe with good thermal-mechanical properties was prepared for the first time by the fluidity modification of UHMWPE and management of thermal-mechanical properties. Compared with those of pure UHMWPE pipe, the Vicat softening temperature (from 128 to 135.2 °C), thermal deformation temperature (from 84.4 to 133.1 °C), bending strength (from 27.3 to 39.8 MPa), and tensile strength (from 20.8 to 25.1 MPa) of OMMT-modified UHMWPE pipe were greatly increased. Our results showed that

(1) screw extrusion molding of UHMWPE was feasible by the modification of UHMWPE;
(2) for the process parameters in this study, the reasonable addition amounts of OMMT and HDPE-g-MAH were 8% and 3%, respectively, for the compatibilizer method, and the reasonable addition amount of KH560 was *w*_KH560_/*w*_OMMT_ = 1% for the coupling agent method;
(3) with the management of thermal-mechanical properties, OMMT-modified UHMWPE pipe with good thermal-mechanical properties was able to be prepared by extrusion for the first time; and
(4) the compatibilizer method of HDPE-g-MAH was slightly more effective than the coupling agent method of KH560.

## Figures and Tables

**Table 1:** Process parameters of screw extrusion molding.

Machine Barrel Temperature/°C			Mold Temperature/°C			Water Jacket	Screw Speed
Zone #1	Zone #2	Zone #3	Zone #1	Zone #2	Zone #3	Temperature/°C	/rpm
120	220	270	245	160	120	20	3

**Table 2:** Thermal and mechanical properties of pure UHMWPE pipe.

Parameter	Value
Vicat Softening Temperature/°C	128
Thermal Deformation Temperature/°C	84.4
Shore Hardness/HD	69
Melting Enthalpy/(J/g)	144.74
Bending Strength/MPa	27.3
Tensile Strength/MPa	20.8

**Table 3:** Mass fraction of each component/%.

No.	1	2	3	4	5	6	7	8
OMMT	5	6	7	7.5	8	8.5	9	10
HDPE-g-MAH	3	3	3	3	3	3	3	3
UHMWPE	59	58	57	56.5	56	55.5	55	54
HDPE	30	30	30	30	30	30	30	30
Other Additives	3	3	3	3	3	3	3	3

**Table 4:** Mass fraction of each component/%.

No.	9	10	11	12	13
OMMT	8	8	8	8	8
HDPE-g-MAH	1	2	3	4	5
UHMWPE	58	57	56	55	54
HDPE	30	30	30	30	30
Other Additives	3	3	3	3	3

**Table 5:** Mass fraction of each component/%.

No.	14	15	16	17	18
OMMT	8	8	8	8	8
KH560	0.04	0.06	0.08	0.10	0.12
UHMWPE	58.96	58.94	58.92	58.90	58.88
HDPE	30	30	30	30	30
Other Additives	3	3	3	3	3
